# A robust microbiome signature for autism spectrum disorder across different studies using machine learning

**DOI:** 10.1038/s41598-023-50601-7

**Published:** 2024-01-08

**Authors:** Lucia N. Peralta-Marzal, David Rojas-Velazquez, Douwe Rigters, Naika Prince, Johan Garssen, Aletta D. Kraneveld, Paula Perez-Pardo, Alejandro Lopez-Rincon

**Affiliations:** 1 Division of Pharmacology, Faculty of Science, Utrecht Institute for Pharmaceutical Sciences, University of Utrecht, Utrecht, The Netherlands; 2 Department of Data Science, Julius Center for Health Sciences and Primary Care, University Medical Center Utrecht, Utrecht, The Netherlands; 3 Global Centre of Excellence Immunology, Danone Nutricia Research, Utrecht, The Netherlands; 4 Department of Neuroscience, Faculty of Science, VU University, Amsterdam, The Netherlands

**Keywords:** Computational biology and bioinformatics, Microbiology, Neuroscience

## Abstract

Autism spectrum disorder (ASD) is a highly complex neurodevelopmental disorder characterized by deficits in sociability and repetitive behaviour, however there is a great heterogeneity within other comorbidities that accompany ASD. Recently, gut microbiome has been pointed out as a plausible contributing factor for ASD development as individuals diagnosed with ASD often suffer from intestinal problems and show a differentiated intestinal microbial composition. Nevertheless, gut microbiome studies in ASD rarely agree on the specific bacterial taxa involved in this disorder. Regarding the potential role of gut microbiome in ASD pathophysiology, our aim is to investigate whether there is a set of bacterial taxa relevant for ASD classification by using a sibling-controlled dataset. Additionally, we aim to validate these results across two independent cohorts as several confounding factors, such as lifestyle, influence both ASD and gut microbiome studies. A machine learning approach, recursive ensemble feature selection (REFS), was applied to 16S rRNA gene sequencing data from 117 subjects (60 ASD cases and 57 siblings) identifying 26 bacterial taxa that discriminate ASD cases from controls. The average area under the curve (AUC) of this specific set of bacteria in the sibling-controlled dataset was 81.6%. Moreover, we applied the selected bacterial taxa in a tenfold cross-validation scheme using two independent cohorts (a total of 223 samples—125 ASD cases and 98 controls). We obtained average AUCs of 74.8% and 74%, respectively. Analysis of the gut microbiome using REFS identified a set of bacterial taxa that can be used to predict the ASD status of children in three distinct cohorts with AUC over 80% for the best-performing classifiers. Our results indicate that the gut microbiome has a strong association with ASD and should not be disregarded as a potential target for therapeutic interventions. Furthermore, our work can contribute to use the proposed approach for identifying microbiome signatures across other 16S rRNA gene sequencing datasets.

## Introduction

Autism spectrum disorder (ASD) is a set of neurodevelopmental disorders which are diagnosed based on behavioural abnormalities, such as deficiencies in social interaction and communication, and repetitive behaviour^1^. Although there is a great variability regarding the prevalence of ASD, it is evident that the number of individuals diagnosed with ASD has been considerably increasing in the last decades specially in countries with high socio-demographic indexes. These growing numbers are not explained solely by the use of newer advanced diagnostic methods, but also by the increase of risk factors for ASD^2,3^. To this date, there are no epidemiological studies investigating ASD prevalence in the Netherlands, however, the number of children with ASD in primary schools in 2018 was 14/1000, similar to prevalence data found in other European countries^4,5^. While worldwide prevalence largely varies, a 4:1 ratio male to female remains consistent across the globe^6^. Both genetic and environmental factors contribute to ASD development^7^, nonetheless, the exact underlying mechanisms that accompany this disorder are yet to be elucidated. Gastrointestinal disturbances such as diarrhea, constipation and abdominal pain are often present in individuals with ASD^8^. In addition, gastrointestinal problems are correlated to a higher degree of ASD severity^9^. Moreover, there are differences in the microbial communities colonizing the gut of ASD individuals when compared to control populations^10^, and some of these changes can alter multiple host’s functions hinting for plausible molecular pathways relevant in ASD^11^. Although abnormal gut microbiota composition has been repeatedly described in ASD, there is no consensus amongst the observed differences in bacterial abundances^12^. In spite of that, multiple lines of associative evidence indicate the importance of these, bidirectional, interactions between microbiota, gut and brain (also referred to as microbiota-gut-brain axis) in ASD and other neurodevelopmental disorders^13^.

Lifestyle, specifically diet, is a major contributing factor when studying human gut microbiome^14,15^. Individuals with ASD commonly lack a diverse diet compared to neurotypical individuals^16^. Siblings are frequently included as control subjects to better control for inter-individual variables like genetic background, household environment and dietary habits^17^. Several studies emphasize the importance and possible role of abnormal gut microbiota in ASD development^18–20^, and how the correction of the bacterial communities living in the gut may be an effective approach for ameliorating intestine- and brain-related problems in ASD^21^. For example, faecal transplantation in Phase-I clinical trial has shown to significantly improve ASD behavioural scores and ASD-associated gastrointestinal symptoms over a time frame of 2 years^22^.

Given the complexity of ASD pathophysiology and the lack of agreement on which gut microbes play an important role in the disorder, this study aims to identify a specific subset of bacteria that is (i) a signature for ASD classification, and (ii) reproducible among other populations. To these aims, we applied a machine learning-based algorithm, named recursive ensemble feature selection (REFS)^23–25^, in three available datasets from the analysis of gut microbiota composition in both ASD and control populations. One dataset including neurotypical siblings as controls was used for feature selection, while the other two datasets, which included unrelated age-matched children as controls, were used for validation^26–28^. Feature selection methods allow us to identify specific traits to predict certain conditions^29^. Thus, this study highlights the advantages of using a machine learning-based method to successfully predict ASD with the minimal number of features, in this case gut bacterial taxa. By analyzing data obtained from distinct cohorts, we suggest that our results are not dependent on other confounding factors such as lifestyle, dietary habits, and geographical region.

## Methods

### Data

Datasets were selected based on (i) availability of raw 16S rRNA gene sequencing data using Illumina, (ii) detailed information regarding the subjects recruited in the study including age, sex, and diagnosis of ASD, among others, (iii) neurotypical siblings for feature selection, and age-matched neurotypical subjects for feature validation as controls, and (iv) subjects between 2 and 7 years old. Raw 16S rRNA gene amplicon sequencing data from the study David et al. was used for feature selection^26^. Paired-end reads 150 bp long from the V4 region of the 16S rRNA gene were sequenced in 60 subjects with autism spectrum disorder (ASD cases), and 57 siblings (controls). Two separate 16S rRNA gene sequencing datasets were used for validation, PRJNA589343 (single reads 250 bp long from V4 region in 77 ASD cases and 50 age-matched controls)^27^ and PRJNA578223 (paired-end reads 300 bp V3–V4 regions in 48 ASD cases and 48 age-matched controls)^28^. See Table 1 for the characteristics of the individuals included in the studies.

**Table 1:** Characteristics of the subjects included in this study.

ASD				NT				
Subjects	M:F	Mean age ± SD	ASD diagnosis	Subjects	M:F	Mean age ± SD	Country	Refs.
60	43:17	5.02 ± 1.59	ADOS	57	27:22^a^	4.56 ± 1.88	USA	^26^
77	59:18	3.21 ± 0.98	CARS	50^b^	39:11	3.58±1.21	China	^27^
48	38:10	5^c^	ADI-R, CGI-S, ABC-I	48^b^	24:24	4^c^	China	^28^

*ASD* autism spectrum disorder, *NT* neurotypical, *M:F* male to female ratio, *SD* standard deviation, *ADOS* autism diagnostic observation schedule, *CARS* childhood autism rating scale, *ADI-R* autism diagnostic interview-revised, *CGI-S* clinical global impression severity of illness scale, *ABC-I* aberrant behavior checklist irritability.

^a^8 missing responses from NT subjects.

^b^Not siblings.

^c^SD not specified.

### Sequence filtering, chimera removal, and taxonomic assignment

Raw sequence reads from David et al.^26^ were processed using the software package DADA2 (version 1.8) under R 4.1.2 environment^30^. The first 10 nucleotides (nt) were trimmed from the forward and reverse reads following DADA2’s recommendation. In addition, reads with more than two expected errors were excluded from the analysis. Consecutively, the reads were independently dereplicated and denoised using DADA2’s default parameters. The resulting forward and reverse reads were merged with a minimum overlap of 20 bases. This resulted in 7160 amplicon sequence variants (ASVs) prior to the removal of chimera sequences. The removal of the chimera sequences resulted in 2040 ASVs, with only 5% of the total reads removed. Taxonomies were assigned to all ASVs using the IDTAXA method from the DECIPHER package^31^. A pretrained classifier based on the SILVA SSU rRNA database (version r138)^31,32^ was used with the IDTAXA method. See Table 2.

**Table 2:** Taxonomy annotation of the 26 selected ASVs using SILVA.

Index	Domain	Phylum	Class	Order	Family	Genus	Species	David et al (2021)	PRJNA578223	PRJNA589343
1	Bacteria	*Proteobacteria*	*Gammaproteobacteria*	*Enterobacterales*	*Enterobacteriaceae*	NA	NA	ASD increased	Yes	Yes
2	Bacteria	*Actinobacteria*	*Actinobacteria*	*Bifidobacteriales*	*Bifidobacteriaceae*	*Bifidobacterium*	NA	ASD decreased	Yes	Yes
3	Bacteria	*Firmicutes*	*Clostridia*	*Eubacteriales*	*Lachnospiraceae*	*Lachnospira*	NA	ASD increased	Yes	Yes
4	Bacteria	*Bacteroidota*	*Bacteroidia*	*Bacteroidales*	*Tannerellaceae*	*Parabacteroides*	NA	ASD increased	Yes	No
5	Bacteria	*Bacteroidota*	*Bacteroidia*	*Bacteroidales*	*Prevotellaceae*	NA	NA	ASD increased	No	Yes
6	Bacteria	*Firmicutes*	*Clostridia*	*Eubacteriales*	*Oscillospiraceae*	*Oscillospira*	NA	ASD increased	No	Yes
7	Bacteria	*Firmicutes*	*Clostridia*	*Eubacteriales*	*Clostridiaceae*	*Sarcina*	NA	ASD increased	Yes	Yes
8	Bacteria	*Firmicutes*	*Clostridia*	*Eubacteriales*	*Lachnospiraceae*	NA	NA	ASD increased	Yes	Yes
9	Bacteria	*Firmicutes*	*Clostridia*	*Eubacteriales*	*Clostridiaceae*	NA	NA	ASD increased	Yes	Yes
10	Bacteria	*Firmicutes*	*Erysipelotrichia*	*Erysipelotrichales*	*Erysipelatoclostridiaceae*	NA	NA	ASD decreased	Yes	Yes
11	Bacteria	*Firmicutes*	*Clostridia*	*Eubacteriales*	*Clostridiaceae*	*Clostridium*	NA	ASD increased	Yes	Yes
12	Bacteria	*Firmicutes*	*Clostridia*	*Eubacteriales*	*Lachnospiraceae*	*Anaerosporobacter*	NA	ASD increased	Yes	Yes
13	Bacteria	*Actinobacteria*	*Coriobacteriia*	*Coriobacteriales*	*Coriobacteriaceae*	*Collinsella*	NA	ASD decreased	Yes	Yes
14	Bacteria	*Firmicutes*	*Clostridia*	*Eubacteriales*	*Clostridiaceae*	*Butyricicoccus*	NA	ASD decreased	Yes	Yes
15	Bacteria	*Firmicutes*	*Clostridia*	*Eubacteriales*	*Lachnospiraceae*	*Lachnospira*	*Eubacterium* *eligens*	ASD decreased	Yes	Yes
16	Bacteria	*Firmicutes*	*Erysipelotrichia*	*Erysipelotrichales*	*Erysipelatoclostridiaceae*	*Erysipelatoclostridium*	NA	ASD decreased	Yes	Yes
17	Bacteria	*Proteobacteria*	*Gammaproteobacteria*	*Enterobacterales*	*Enterobacteriaceae*	NA	NA	ASD increased	Yes	Yes
18	Bacteria	*Firmicutes*	*Clostridia*	*Eubacteriales*	*Lachnospiraceae*	NA	NA	ASD increased	No	No
19	Bacteria	*Firmicutes*	*Clostridia*	*Eubacteriales*	*Lachnospiraceae*	*Lachnospiraceae* UCG-004	NA	ASD decreased	Yes	Yes
20	Bacteria	*Firmicutes*	*Clostridia*	*Eubacteriales*	*Clostridiaceae*	*Clostridium*	NA	ASD increased	Yes	Yes
21	NA	NA	NA	NA	NA	NA	NA	ASD increased	Yes	Yes
22	Bacteria	*Firmicutes*	*Tissierellia*	*Tissierellales*	*Peptoniphilaceae*	*Murdochiella*	NA	ASD decreased	No	Yes
23	Bacteria	*Firmicutes*	*Clostridia*	*Eubacteriales*	*Lachnospiraceae*	NA	NA	ASD increased	No	No
24	Bacteria	*Proteobacteria*	*Gammaproteobacteria*	*Enterobacterales*	*Enterobacteriaceae*	NA	NA	ASD decreased	Yes	Yes
25	Bacteria	NA	NA	NA	NA	NA	NA	ASD increased	No	No
26	Bacteria	*Firmicutes*	*Clostridia*	*Eubacteriales*	*Clostridiaceae*	*Clostridium*	NA	ASD decreased	Yes	Yes

Information includes index for feature importance based on REFS, whether the differential abundance of the selected ASVs was increased or decrease in ASD cases compared to controls of the discovery dataset (David et al.^26^), and whether each ASV was found in the validation datasets Zou et al.^28^ and Ding et al.^27^—PRJNA578223 and PRJNA589343, respectively.

Raw sequence reads from PRJNA589343^27^ were processed following the aforementioned procedure. The set filtering parameters included no truncation and a maximum of two expected errors per read, and the lack of a merging step as this dataset consists of single-ended reads (only forward reads). This resulted in 2030 ASVs after removal of the chimera sequences. Raw sequence reads from PRJNA578223^28^ were processed in a similar manner with the following filtering parameters: truncate forward and reverse reads to 290 and 220 nt, respectively; and remove all reads with more than two expected errors. This resulted in 18,758 ASVs after removal of chimera sequences.

### Feature selection and validation

We used REFS^23–25^, a method applied for discovering biomarkers, to determine which ASVs are appropriate for differentiating ASD cases from controls. The ensemble is composed by 8 classifiers from the sci-kit learn toolbox^33^: Stochastic Gradient Descent (SGD), Support Vector Machine classifier (SVC), gradient boosting, random forest, logistic regression, passive aggressive classifier, ridge classifier and bagging. It is known that working with a small number of samples can cause overfitting, to avoid this problem, REFS uses nested-cross validation in a tenfold cross-validation scheme, a proven solution to produce more accurate and unbiased results regarding the number of samples^34^. Prior to feature selection, the data (matrix containing ASVs’ counts) was normalized using scikit-learn’s Z-score algorithm^33^. Each cycle of REFS removed the 20% least important ASVs, until only one feature was left. To prevent method’s randomization from negatively affecting results, this process was concurrently run 30 times. For each run, performance metrics including averages and variances were calculated. The reduced features were selected based on the best performing cycle of the best run. To avoid bias selection of the ASVs, we performed a validation process similarly as it has been previously described in previous studies^23–25^. This process applies 5 different classifiers that do not belong to the ensemble from the sci-kit learn toolbox^33^: AdaBoost, Extra Trees, KNeighbors, Multi-Layer Perceptron (MLP), and Least Absolute Shrinkage and Selection Operator plus iterative process using Cross-Validation (LassoCV). The accuracy given by the average of the five classifiers in a nested tenfold cross-validation gives us an area under the curve (AUC) that estimates the power of a discriminant test, being more successful with values close to 1.0 which reflects an excellent accuracy^35^.

**Figure 1:**
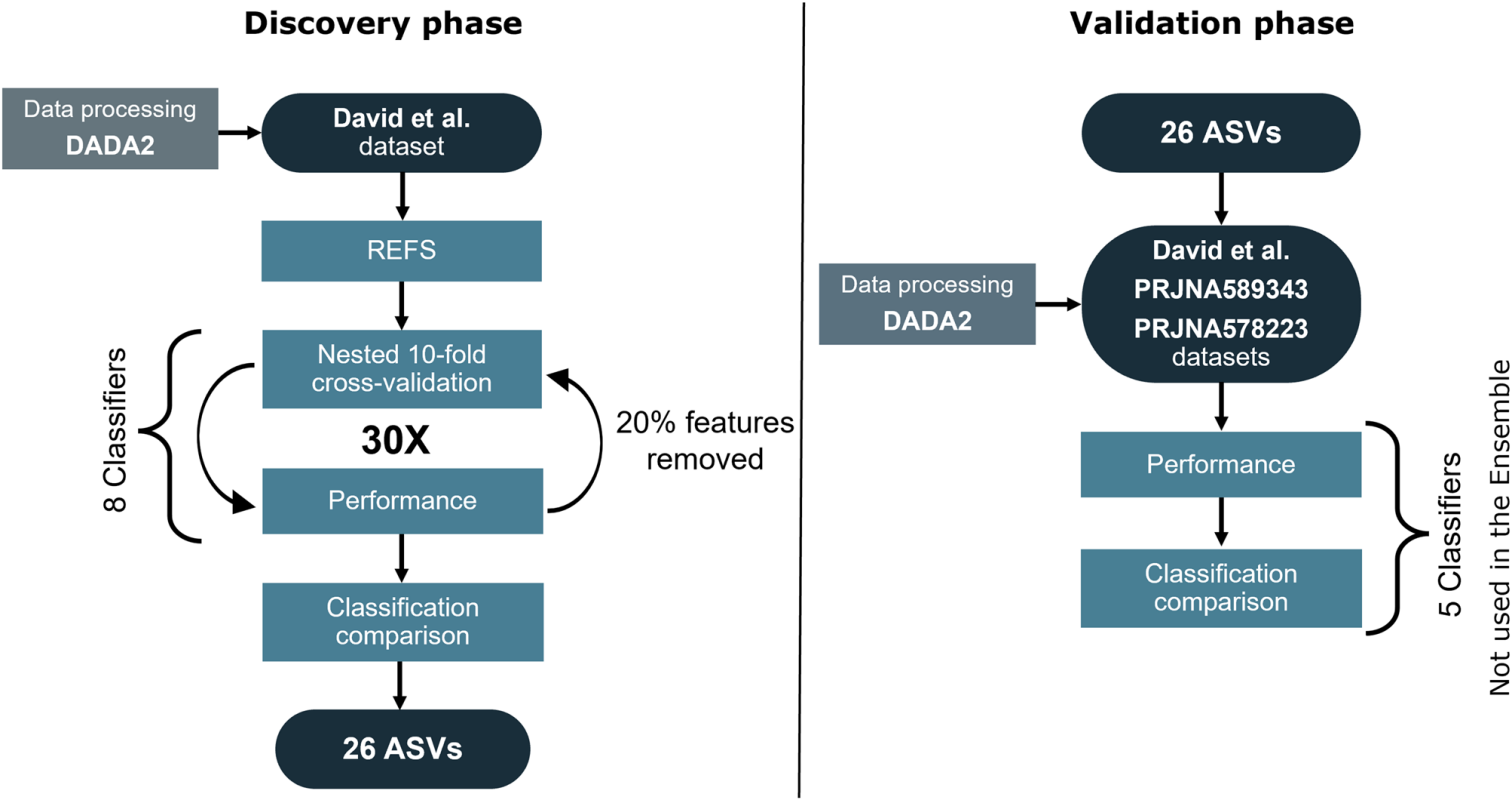
Bioinformatic pipeline to select the optimal number of ASVs associated to ASD phenotype by applying REFS to a 16S rRNA gene sequencing dataset (discovery phase), and validation of the selected set of ASVs across different cohorts (valiation phase)^26–28^.

The selected top scoring ASVs, were then validated in PRJNA589343 and PRJNA578223 datasets^27,28^. After processing the 16s rRNA raw sequences using DADA2, top scoring ASVs were extracted from the resulting ASVs of the validation datasets. The reads’ length in both validation datasets were 250 bp and 300 bp, respectively. As the selected ASVs of the discovery dataset were shorter (150 bp), ASVs were counted in the validation datasets when an exact match was found. Abundance data of all matching ASVs was added together and treated as one during validation. The resulting filtered datasets were tested using a tenfold cross-validation with the 5 classifiers different from the classifiers used for the ensemble, and AUC of the receiver operating characteristic (ROC) curves were calculated. Whether the selected 26 ASVs were present or not in the validation datasets can be found in Table 2. For an overview of the methodology, see Fig. 1.

### Differential abundance

To determine differential abundance, the reduced discovery dataset was normalized using scikit-learn’s StandardScaler scaling algorithm^33^. Then, differential abundances for the discovery dataset for each selected ASVs were plotted as a heat-map comparing ASD cases and controls. See Supplementary Figure 1. Likewise, normalized differential abundances were plotted for the identified ASVs found on each validation dataset. See Supplementary Figs. 2 and 3. Heat-maps were created using the python script heatmap.py.

## Results

### Feature selection and validation of case-control cohorts for ASD

**Figure 2:**
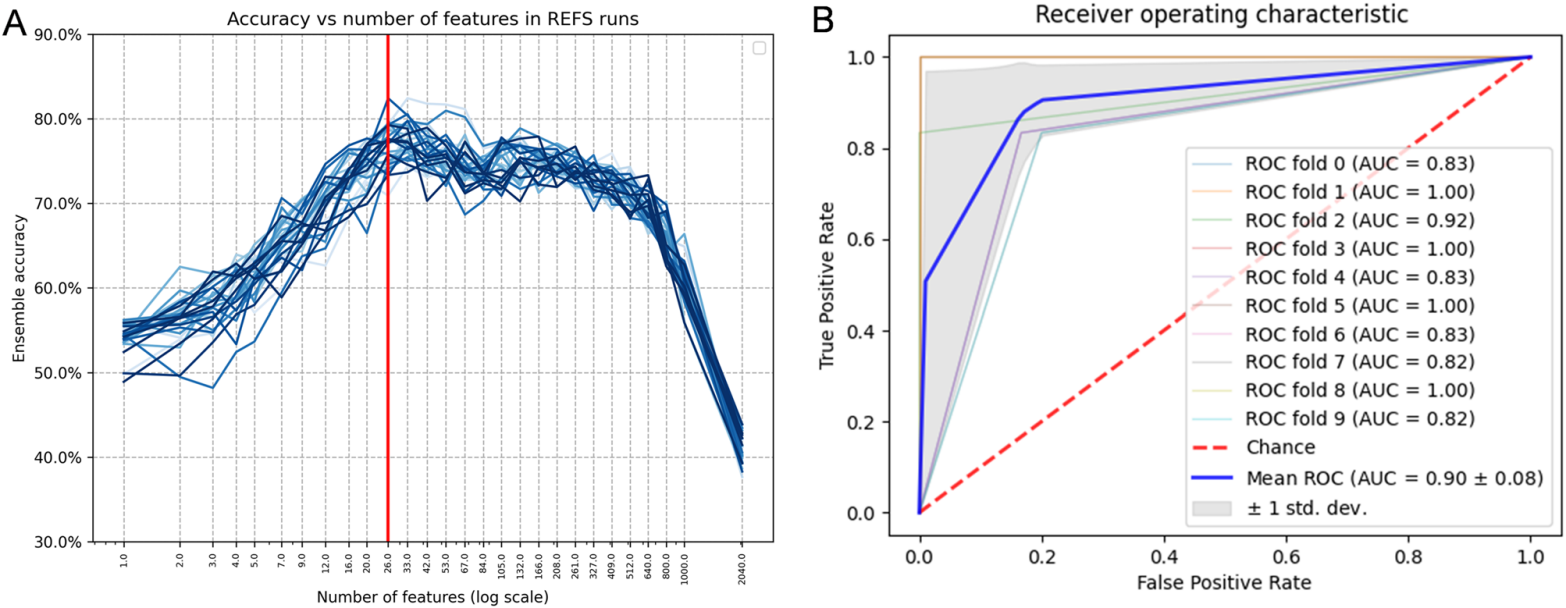
(**A**) Optimal number of features, ASVs, for ASD classification applying REFS to the discovery dataset^26^. (**B**) ROC curve of the 26 selected features, ASVs, in the best performing classifier, Multi-Layer Perceptron (MLP), in REFS for the discovery dataset^26^.

For feature selection, we used a dataset of 16S rRNA gene sequences from 117 subjects (60 ASD cases and 57 siblings)^26^. After applying REFS, features were reduced from 2040 ASVs (processed 16rRNA raw sequences obtained with DADA2) to 26 ASVs as the optimal number of features to distinguish between ASD cases and controls (see Fig. 2A). AUC of ROC curves was used for evaluating the diagnostic accuracy of each classifier. Mean classification accuracy in a tenfold cross-validation method increases when using the set of 26 reduced features compared to the 2040 processed features (average AUC = 0.816 and average AUC = 0.41, respectively). See results in Table 3.

**Table 3:** Accuracy of nested cross-validation (tenfold cross-validation) classifiers used for REFS on the discovery dataset^26^.

Classifier	26 Features		2040 Features	
	Average AUC	SD	Average AUC	SD
AdaBoostClassifier	0.720	0.010	0.390	0.140
Extra trees	0.780	0.080	0.340	0.160
KNeighbors	0.790	0.100	0.420	0.070
MLP	0.900	0.080	0.410	0.100
LassoCV	0.890	0.090	0.500	0.000
Average	0.816	0.072	0.410	0.090

*REFS* recursive ensemble feature selection, *AUC* area under the curve, *SD* standard deviation, *MLP* multi-layer perceptron, *LassoCV* least absolute shrinkage and selection operator plus iterative process using cross-validation.

**Figure 3:**
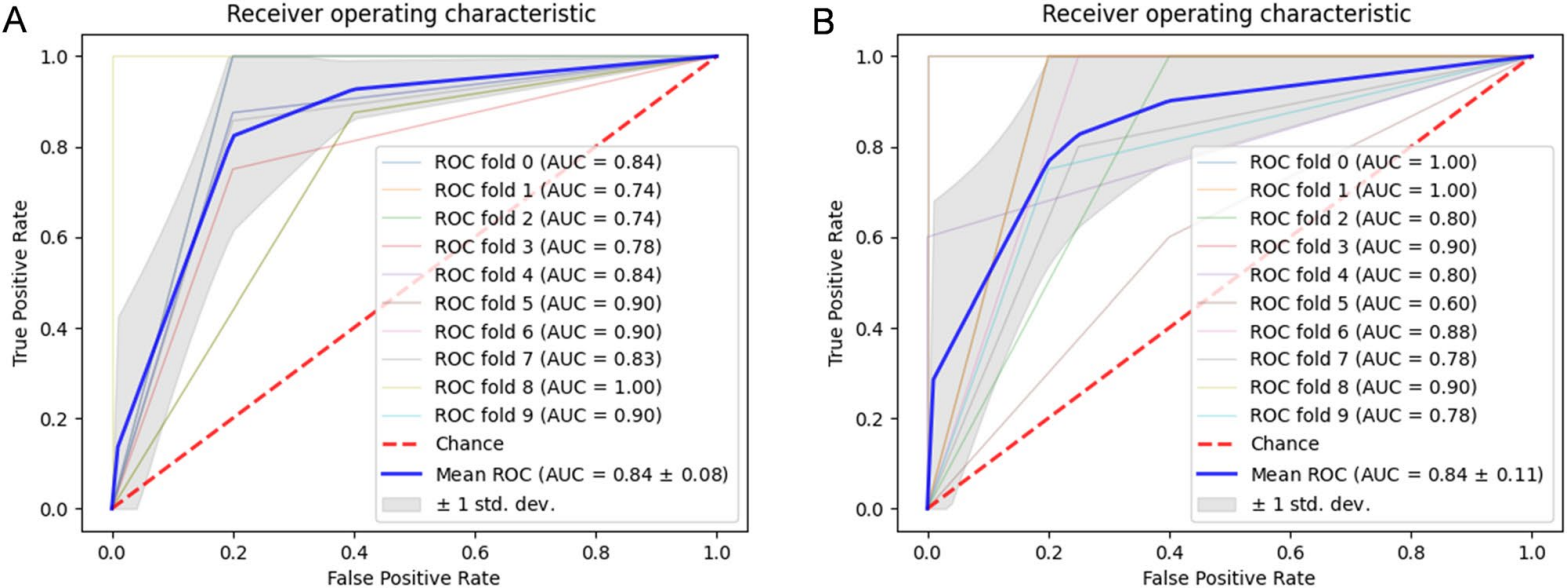
(**A**,**B**) ROC curves of the 26 selected features, ASVs, in the best performing classifier, Extra Trees, in REFS for both validation datasets^27,28^, respectively.

For feature validation, we used 16S rRNA gene sequencing data from two age-matched cohorts with a total of 223 samples (125 ASD cases and 98 controls)^27,28^. We evaluated the mean AUC of the ROC curve of the previously identified 26 features employing the validation datasets in a tenfold cross-validation testing five classifiers using REFS. Although not all 26 sequences were found in both validation datasets, 22 and 20 ASVs were found, respectively^27,28^ (see Table 2). In comparison to the classification accuracy mean of the discovery set (average AUC = 0.816), the scores of the validation sets resulted in good diagnostic accuracy (average AUC=0.748 and average AUC=0.74, respectively; see results in Table 4). Specifically looking at the classifier with the best diagnostic accuracy, MLP for the discovery dataset and Extra Trees for both validation datasets, we reported an AUC=0.90 for the discovery cohort, see Fig. 2B, and an AUC=0.84 for both validation datasets, see Fig. 3.

**Table 4:** Accuracy of a set of 26 features selected from the discovery dataset^26^ in nested cross-validation (tenfold cross-validation) classifiers in the validation sets^27,28^.

Classifier	Validation set^27^		Validation set^28^	
	Average AUC	SD	Average AUC	SD
Adaboost	0.770	0.080	0.830	0.080
Extra Trees	0.840	0.080	0.840	0.110
KNeighbors	0.680	0.090	0.700	0.150
MLP	0.740	0.120	0.720	0.080
LassoCV	0.710	0.100	0.610	0.100
Average	0.748	0.094	0.740	0.104

*AUC* area under the curve; *SD* standard deviation; *MLP* multi-layer perceptron, *LassoCV* least absolute shrinkage and selection operator plus iterative process using cross-validation.

### Differential abundances of 26 specific bacterial taxa to distinguish between ASD cases and controls

To better understand the role of gut microbiota composition in ASD, we investigated the differential abundances and the taxonomy of the 26 identified features, the 26 ASVs, in the discovery dataset^26^. See Table 2 for the assigned taxonomies to the selected ASVs, and Supplementary Fig. 1 for differential abundances.

At phylum level, we identified 17 ASVs belonging to the phylum *Firmicutes*, 3 ASVs to *Proteobacteria*, 2 ASVs to *Bacteroidota*, 2 ASVs to *Actinobacteria*, and 2 ASVs were not assigned to any phyla. At a family level, 7 ASVs belong to *Lachnospiraceae*, 6 ASVs to *Clostridiaceae*, 3 ASVs to *Enterobacteriaceae*, 2 ASVs to *Erysipelatoclostridiaceae*, single ASVs were assigned to *Bifidobacteriaceae*, *Prevotellaceae*, *Tannerellaceae*, *Oscillospiraceae*, *Coribacteriaceae* and *Peptoniphilaceae*, and 2 ASVs were not assigned to any family group. At a genus level, 11 ASVs were not assigned to any genera, 3 ASVs belong to *Clostridium*, 2 ASVs to *Lachnospira*, and single ASVs to *Bifidobacterium*, *Parabacteroides*, *Oscillospira*, *Sarcina*, *Anaerosporobacter*, *Collinsella*, *Butyricicoccus*, *Lachnospiraceae* UCG-004, *Erysipelatoclostridium* and *Murdochiella*. Only one ASVs was assigned to species level, *Eubacterium eligens*.

Both genera belonging to *Actinobacteria* phylum, *Bifidobacterium* and *Collinsella*, were decreased in ASD cases compared to controls. On the contrary, the two bacterial taxa belonging to *Bacteroidota* phylum, *Prevotellaceae* and *Parabacteroides*, were increased in ASD cases when compared to controls. Among the 3 different ASVs belonging to the *Proteobacteria* phylum, all of them assigned to the *Enterobacteriaceae* family, 2 of them were increased in ASD cases while the other one was increased in controls. The latter ASV-associated bacterium, was not found in any ASD subject. Within the ASVs from the phylum *Firmicutes*, we reported lower abundances of *Erysipelatoclostridiaceae*, *Murdochiella*, *Butyricicoccus*, *Clostridium*, *Lachnospiraceae* UCG-004, and *Eubacterium eligens*. Within the same phylum, increased differential abundances of bacterial taxa represented by the selected ASVs included *Lachnospiraceae*, *Clostridium*, *Sarcina*, *Anaerosporobacter*, and *Oscillospira*. In addition, 3 ASVs assigned to *Lachnospiraceae* and *Oscillospira* were not present in any control subject.

## Discussion

Recently, the use of machine learning-based techniques is becoming more popular to study complex systems, and that has been the case for ASD. Artificial intelligence has been mostly applied to overcome limitations of traditional diagnostic methods^37,38^. If we also consider its potential use for microbiome studies in human health^39^, implementing machine learning to study the relationship between gut dysbiosis and ASD can give us valuable insights to understand the disorder, to better diagnoses, and to develop plausible therapies.

Microbiome analyses have multiple challenges that compromise the interpretation and reproducibility of the results across studies. Besides the importance of defining well-standardized methods to collect, store, process, and sequence the samples, it is important to agree on a consensual approach to analyze the data in order to draw correct conclusions^40^. Several publications have investigated the effect of confounding effects in microbiome outcomes in ASD, as well as discrepancies based on the method to process the sequencing data like clustering the data in operational taxonomic units (OTUs) rather than ASVs^41–44^. The present study, using the machine learning approach REFS, identified a specific set of bacterial taxa from ASV taxonomic annotation that is sufficient to optimally differentiate between ASD cases and controls in a published sibling-controlled dataset^26^. In addition, we demonstrated that this set of bacterial taxa can distinguish between ASD and control populations in two independent published datasets^27,28^, indicating the robustness of the method.

We analyzed 16S rRNA gene sequencing data using the aforementioned machine-learning approach based on integrative analysis allowing to study the compositional nature of the gut microbiome. A proper statistical practice is essential for the correct interpretation of the analysis of microbiome data as using traditional statistical methods often present assumptions and biases^45,46^. Looking into literature, approaches that integrate feature selection and cross-validation strategies provide a good predictive tool by discriminating between two phenotypes with the least number of relevant features^47,48^. REFS has been successfully employed previously in other medical research studies using microRNA expression and messenger RNA expression data^49,50^, indicating its large potential being applied to other kinds of biological data.

Besides reducing the relevant features for ASD classification from 2040 ASVs to 26 ASVs using one dataset, we also tested how important these ASVs are for ASD classification, thus their application as a predictive tool, by validating them in other two independent datasets. Overall, our results showed good prediction accuracy means. For the discovery dataset, the best-performing classifier reported an AUC value of 0.90 using the dataset from David et al.^26^. During the validation phase, the best-performing classifier showed an AUC value of 0.84 for both datasets Ding et al.^27^ and Zou et al.^28^ when the 26 selected ASVs were used for ASD status classification. According to Šimundić^35^, these results indicate a highly accurate predictive method for discriminating ASD phenotype using 16S rRNA gene sequenced data despite of the expected high variability of the included populations in the study as they have different geographical regions, and most likely different lifestyles. Using our proposed approach to further analyze existing microbiome data or new data from longitudinal-designed studies including ASD and control populations will be valuable to better understand, and corroborate, the role of these 26 selected bacterial taxa in ASD development and progression.

A recent study by Yap et al. did not find grounds for an associative link between the gut microbiota and ASD diagnosis, implying that differences in the gut microbiota composition of ASD subjects are solely a consequence of ASD-related feeding behaviour^51^. However, some research has been done regarding the potential use of gut microbiome data for ASD diagnosis making use of machine learning approaches. In three compelling meta-analyses, Wu et al., Chavira et al. and Pietrucci et al. processed gut microbiome data across different existing ASD studies concluding that gut dysbiosis is associated to ASD, nonetheless, other factors such as sex, age, geographical region and lifestyle cannot be ignored when studying the role of intestinal microbes in ASD^52–54^. All meta-analyses used a fivefold cross-validation approach for machine learning classification on taxonomic annotation data, but the number and type of classifiers differed between the studies. In Chavira et al.^53^, they examined how taxonomic resolution impact predictive accuracy concluding that the higher the taxonomic resolution is, the better the models’ performance is. Additionally, Pietrucci et al.^54^ looked at the importance of the control groups for ASD classification using three different classifiers. Their results reported better accuracy when unrelated control groups were used for ASD classification. It can be explained by the fact that siblings have more similar microbiomes than unrelated individuals as they share more alike environment, lifestyle and genetics^55^. While in these three studies a collective analysis was performed to study differential abundances of the intestinal microorganisms and the most relevant features for ASD classification, we applied feature selection in a sibling-control cohort and tested the selected features in two independent cohorts showing that other confounding factors are not interfering the reliability of the results. Moreover, the input data in our approach are ASVs rather than OTUs or taxonomic annotation allowing us to identify specific bacterial taxa with one-nucleotide difference^56^.

Many of the studies investigating the influence of the gut bacteria in ASD pathophysiology, targeted microbiota composition by analyzing 16S rRNA gene sequencing data, measuring relative abundances of the present bacteria, and then searching for correlations between each individual bacterial taxa with other ASD-related traits. A serious limitation of using conventional statistical strategies in this type of compositional and high-dimensional data can lead to erroneous outcomes^57,58^. A possible way to overcome this problem is the use of multivariate analysis rather than univariate analysis, thus our study uses a multivariate, multidataset approach. Here, we identified 26 ASVs that, as a community, can separate ASD phenotype from neurotypical controls.

Moreover, looking at the differential abundances between ASD cases and controls in the discovery dataset, we also observed noticeable changes between ASD and control cases. In line with several studies that reported lower faecal *Bifidobacteria* abundance in children with ASD compared to neurotypical children^59–63^, we also identified an ASV annotated as *Bifidobacterium* that was decreased in ASD cases compared to controls. One of the plausible pathways explaining the involvement of gut bacterial imbalance in ASD is metabolism. In a study, germ-free mice received human faecal microbiota transplantation using samples from either children with ASD or typically-developed children. The results showed alterations in tryptophan metabolism, and *Bifidobacteria* changes correlated with differently abundant metabolites from derivatives of tryptophan metabolisms^64^. Furthermore, dietary interventions either with *Bifidobacterium* strains or other bacterial strains and/or compounds that increase *Bifidobacteria* levels in the gut have shown to improve, not only intestinal outcomes, but also ASD severity^65,66^.

On the other hand, we observed increased bacterial taxa in ASD phenotype including *Clostridia*, *Sarcina* and *Parabacteroides*, among others, that have been also found to be increased in children with ASD^10,59,62,67–69^. Interestingly, one of the aforementioned meta-analysis of gut microbiome data in ASD, also discovered a relevant feature for ASD classification belonging to the *Sarcina* genus, being increased in ASD cases compared to control cases^53^. Although commensal *Clostridia* in the gut help to develop and maintain an intestinal homeostatic state and accordingly a balanced functioning of host’s biological processes, abnormal levels of some members of this genus have been widely linked to health problems including neurodevelopmental disorder susceptibility^70^. Molecular pathways by which *Clostridia* can influence ASD involve metabolic, immunological and physiological processes^71,72^. Additionally, antibiotic usage against these bacteria has shown beneficial effects in behavioural parameters of ASD studies^73^. However, there is no consensus among other studies indicating that the beneficial effects stopped once the antibiotic intervention was finalized, and furthermore pre- and post-natal antibiotic use has been linked to ASD development in several studies^11,74^. In our study we also observed a reduction of ASVs associated to *Butyricicoccus* and *Eubacterium eligens* known to exert beneficial effects by modulating immune response and producing health-promoting compounds as short-chain fatty acids^75–77^. Recently, in a propionic acid rat model for ASD it was shown that therapeutic interventions like *Bifidobacterium longum* or faecal transplantation ameliorated gut dysbiosis restoring *Butyricicoccus* levels^78^. Taking into account all these associative evidence, targeting the gut microbiome with dietary interventions might improve ASD-related complaints. However, we need to comprehend the biological meaning of bacterial changes in ASD to check the added value of specific bacterial taxa as new diagnostic tool, and the use of gut microbiome modulation for improving ASD-related complaints.

A limitation of the current study is the different ASV length after processing the raw 16S rRNA gene sequencing data for each dataset. Because of the selected ASVs are shorter than the ASVs from both validation datasets, we cannot make a clear association between the specific bacterial taxa of the discovery dataset and the validation datasets. Most of the 26 selected ASV were taxonomically assigned to family or genus level, nevertheless, the amount of bacterial species and strains comprised in a single family or genus is remarkably high^79^. When the 26 ASVs were matched with the validation ASVs, there were multiple cases that more than one validation ASV contained a selected ASV indicating that different but closely related bacteria share that specific sequence. In addition, confounding factors that are known to influence the gut microbiota should be further explored in the context of ASD. For instance, evidence show that sex differences potentially have an important role in ASD patophysiology and support the observed male-sex bias^80,81^, and specifically in analysis targeting the gut microbiota^82^.

## Conclusions

Overall our results demonstrate a strong microbiome signature for the classification of ASD in three independent cohorts. Our approach identified 26 features, bacterial taxa, that distinguish ASD cases from control cases with high accuracy. The method that we propose overcomes problems of bias and overfitting results by selecting the smallest number of relevant features identifying ASD status using several classification algorithms that rank the features differently. The discovery of a robust set of bacterial taxa associated to ASD phenotype can potentially be used for diagnostic purposes, and it might provide new insights into plausible molecular mechanisms of the gut-brain axis in ASD. However, further studies should aim to understand the biological significance of these specific bacteria in ASD pathophysiology, and additional data such as metabolic function of the gut microbiome may be of great importance to pursue this line of investigation.

### Supplementary Information

Supplementary Figures.

## Data Availability

Data from David et al.^26^ was obtained from http://files.cgrb.oregonstate.edu/David_Lab/ASD_study1/. Data from Ding et al.^27^ and Zou et al.^28^ was obtained from the GeneBank Sequence Read Archive^36^ using sra-toolkit-2.11.3 (accession numbers PRJNA589434 and PRJNA578223, respectively). Code used to run the analyses is available on Github https://github.com/steppenwolf0/MicrobiomeREFS.git.

## Abbreviations

ABC-I: Aberrant behavior checklist irritability
ADI-R: Autism diagnostic interview-revised
ADOS: Autism diagnostic observation schedule
ASD: Autism spectrum disorder
ASVs: Amplicon sequence variants
AUC: Area under the curve
bp: Base pair
CARS: Childhood autism rating scale
CGI-S: Clinical global impression severity of illness scale
KNN: k-Nearest neighbors
LassoCV: Least absolute shrinkage and selection operator plus iterative process using cross-validation
M:F: Male to female ratio
MLP: Multi-layer perceptron
NT: Neurotypical
nt: Nucleotide
OTUs: Operational taxonomic units
REFS: Recursive ensemble feature selection
ROC: Receiver operating characteristic
SD: Standard deviation
SGD: Stochastic gradient descent
SVC: Support vector classifiers

## References

[1] Happé F, Frith U (2020). Annual research review: Looking back to look forward-changes in the concept of autism and implications for future research. J. Child Psychol. Psychiatry.

[2] Arango C (2021). Risk and protective factors for mental disorders beyond genetics: An evidence-based atlas. World Psychiatry.

[3] Solmi M (2022). Incidence, prevalence, and global burden of autism spectrum disorder from 1990 to 2019 across 204 countries. Mol. Psychiatry.

[4] van der Gaag RJ (2018). The Netherlands and Autism.

[5] Chiarotti F, Venerosi A (2020). Epidemiology of autism spectrum disorders: A review of worldwide prevalence estimates since 2014. Brain Sci..

[6] Zeidan J (2022). Global prevalence of autism: A systematic review update. Autism Res..

[7] Chaste P, Leboyer M (2022). Autism risk factors: Genes, environment, and gene–environment interactions. Dialog. Clin. Neurosci..

[8] Madra M, Ringel R, Margolis KG (2020). Gastrointestinal issues and autism spectrum disorder. Child Adolesc. Psychiatr. Clin. N. Am..

[9] Lefter R, Ciobica A, Timofte D, Stanciu C, Trifan A (2019). A descriptive review on the prevalence of gastrointestinal disturbances and their multiple associations in autism spectrum disorder. Medicina.

[10] Iglesias-Vázquez L, Van Ginkel Riba G, Arija V, Canals J (2020). Composition of gut microbiota in children with autism spectrum disorder: A systematic review and meta-analysis. Nutrients.

[11] Fattorusso A, Di Genova L, Dell’Isola GB, Mencaroni E, Esposito S (2019). Autism spectrum disorders and the gut microbiota. Nutrients.

[12] Peralta-Marzal LN (2021). The impact of gut microbiota-derived metabolites in autism spectrum disorders. Int. J. Mol. Sci..

[13] Cryan JF (2019). The microbiota-gut-brain axis. Physiol. Rev..

[14] Moschen AR, Wieser V, Tilg H (2012). Dietary factors: Major regulators of the gut’s microbiota. Gut Liver.

[15] David LA (2014). Diet rapidly and reproducibly alters the human gut microbiome. Nature.

[16] Rashid A, Iftikhar N, Badar SA, Masood F, Rehman I (2021). Factors influencing food selectivity and food preferences of children with autism spectrum disorder. J. Pharm. Res. Int..

[17] Krajmalnik-Brown R, Lozupone C, Kang D-W, Adams JB (2015). Gut bacteria in children with autism spectrum disorders: Challenges and promise of studying how a complex community influences a complex disease. Microb. Ecol. Health Dis..

[18] Vuong HE, Hsiao EY (2017). Emerging roles for the gut microbiome in autism spectrum disorder. Biol. Psychiatry.

[19] Hughes HK, Rose D, Ashwood P (2018). The gut microbiota and dysbiosis in autism spectrum disorders. Curr. Neurol. Neurosci. Rep..

[20] Tataru C (2021). Longitudinal study of stool-associated microbial taxa in sibling pairs with and without autism spectrum disorder. ISME Commun..

[21] Yang Y, Tian J, Yang B (2018). Targeting gut microbiome: A novel and potential therapy for autism. Life Sci..

[22] Kang D-W (2019). Long-term benefit of microbiota transfer therapy on autism symptoms and gut microbiota. Sci. Rep..

[23] Lopez-Rincon A, Martinez-Archundia M, Martinez-Ruiz GU, Schoenhuth A, Tonda A (2019). Automatic discovery of 100-MiRNA signature for cancer classification using ensemble feature selection. BMC Bioinform..

[24] Kamphorst K (2023). Predictive factors for allergy at 4–6 years of age based on machine learning: A pilot study. PharmaNutrition.

[25] Blankestijn JM (2023). Classifying asthma control using salivary and fecal bacterial microbiome in children with moderate-to-severe asthma. Pediatr. Allergy Immunol..

[26] David MM (2021). Children with autism and their typically developing siblings differ in amplicon sequence variants and predicted functions of stool-associated microbes. Msystems.

[27] Ding X (2020). Gut microbiota changes in patients with autism spectrum disorders. J. Psychiatr. Res..

[28] Zou R (2020). Changes in the gut microbiota of children with autism spectrum disorder. Autism Res..

[29] Zhou Y-H, Gallins P (2019). A review and tutorial of machine learning methods for microbiome host trait prediction. Front. Genet..

[30] Callahan BJ (2016). Dada2: High-resolution sample inference from illumina amplicon data. Nat. Methods.

[31] Murali A, Bhargava A, Wright ES (2018). Idtaxa: A novel approach for accurate taxonomic classification of microbiome sequences. Microbiome.

[32] Quast C (2012). The silva ribosomal rna gene database project: Improved data processing and web-based tools. Nucleic Acids Res..

[33] Pedregosa F (2011). Scikit-learn: Machine learning in python. J. Mach. Learn. Res..

[34] Vabalas A, Gowen E, Poliakoff E, Casson AJ (2019). Machine learning algorithm validation with a limited sample size. PLoS ONE.

[35] Šimundić A-M (2009). Measures of diagnostic accuracy: Basic definitions. EJIFCC.

[36] Leinonen R, Sugawara H, Shumway M, Collaboration INSD (2010). The sequence read archive. Nucleic Acids Res..

[37] Raj S, Masood S (2020). Analysis and detection of autism spectrum disorder using machine learning techniques. Procedia Comput. Sci..

[38] Hossain MD, Kabir MA, Anwar A, Islam MZ (2021). Detecting autism spectrum disorder using machine learning techniques: An experimental analysis on toddler, child, adolescent and adult datasets. Health Inf. Sci. Syst..

[39] Marcos-Zambrano LJ (2021). Applications of machine learning in human microbiome studies: A review on feature selection, biomarker identification, disease prediction and treatment. Front. Microbiol..

[40] Schloss PD (2018). Identifying and overcoming threats to reproducibility, replicability, robustness, and generalizability in microbiome research. MBio.

[41] Shin J (2016). Analysis of the mouse gut microbiome using full-length 16s rrna amplicon sequencing. Sci. Rep..

[42] Nearing JT (2022). Microbiome differential abundance methods produce different results across 38 datasets. Nat. Commun..

[43] Chiarello M, McCauley M, Villéger S, Jackson CR (2022). Ranking the biases: The choice of otus vs asvs in 16s rrna amplicon data analysis has stronger effects on diversity measures than rarefaction and otu identity threshold. PLoS ONE.

[44] Jeske JT, Gallert C (2022). Microbiome analysis via otu and asv-based pipelines—A comparative interpretation of ecological data in wwtp systems. Bioengineering.

[45] Tsilimigras MC, Fodor AA (2016). Compositional data analysis of the microbiome: Fundamentals, tools, and challenges. Ann. Epidemiol..

[46] Quinn TP, Erb I, Richardson MF, Crowley TM (2018). Understanding sequencing data as compositions: An outlook and review. Bioinformatics.

[47] Ditzler G, Morrison JC, Lan Y, Rosen GL (2015). Fizzy: Feature subset selection for metagenomics. BMC Bioinform..

[48] Pasolli E, Truong DT, Malik F, Waldron L, Segata N (2016). Machine learning meta-analysis of large metagenomic datasets: Tools and biological insights. PLoS Comput. Biol..

[49] Lopez-Rincon A (2020). Machine learning-based ensemble recursive feature selection of circulating mirnas for cancer tumor classification. Cancers.

[50] Lopez-Rincon, A. *et al.* Modelling asthma patients’ responsiveness to treatment using feature selection and evolutionary computation. In *International Conference on the Applications of Evolutionary Computation (Part of EvoStar)* 359–372 (Springer, 2021).

[51] Yap CX (2021). Autism-related dietary preferences mediate autism-gut microbiome associations. Cell.

[52] Wu T (2020). Potential of gut microbiome for detection of autism spectrum disorder. Microb. Pathog..

[53] Chavira A, Wang EH-J, Mills RH (2022). Meta-analysis of the autism gut microbiome identifies factors influencing study discrepancies and machine learning classification. BioRxiv..

[54] Pietrucci D (2022). Machine learning data analysis highlights the role of parasutterella and alloprevotella in autism spectrum disorders. Biomedicines.

[55] Gacesa R (2022). Environmental factors shaping the gut microbiome in a dutch population. Nature.

[56] Callahan BJ, McMurdie PJ, Holmes SP (2017). Exact sequence variants should replace operational taxonomic units in marker-gene data analysis. ISME J..

[57] Gilbert JA, Lynch SV (2019). Community ecology as a framework for human microbiome research. Nat. Med..

[58] Hernández Medina R (2022). Machine learning and deep learning applications in microbiome research. ISME Commun..

[59] Finegold SM (2010). Pyrosequencing study of fecal microflora of autistic and control children. Anaerobe.

[60] Adams JB, Johansen LJ, Powell LD, Quig D, Rubin RA (2011). Gastrointestinal flora and gastrointestinal status in children with autism-comparisons to typical children and correlation with autism severity. BMC Gastroenterol..

[61] Wang L (2011). Low relative abundances of the mucolytic bacterium *Akkermansia muciniphila* and *Bifidobacterium* spp. in feces of children with autism. Appl. Environ. Microbiol..

[62] De Angelis M (2013). Fecal microbiota and metabolome of children with autism and pervasive developmental disorder not otherwise specified. PLoS ONE.

[63] Coretti L (2018). Gut microbiota features in young children with autism spectrum disorders. Front. Microbiol..

[64] Xiao L (2021). Fecal microbiome transplantation from children with autism spectrum disorder modulates tryptophan and serotonergic synapse metabolism and induces altered behaviors in germ-free mice. Msystems.

[65] Grimaldi R (2018). A prebiotic intervention study in children with autism spectrum disorders (ASDS). Microbiome.

[66] Shaaban SY (2018). The role of probiotics in children with autism spectrum disorder: A prospective, open-label study. Nutr. Neurosci..

[67] Wang M (2019). Alterations in gut glutamate metabolism associated with changes in gut microbiota composition in children with autism spectrum disorder. Msystems.

[68] Parracho HM, Bingham MO, Gibson GR, McCartney AL (2005). Differences between the gut microflora of children with autistic spectrum disorders and that of healthy children. J. Med. Microbiol..

[69] Luna RA (2017). Distinct microbiome-neuroimmune signatures correlate with functional abdominal pain in children with autism spectrum disorder. Cell. Mol. Gastroenterol. Hepatol..

[70] Ding HT, Taur Y, Walkup JT (2017). Gut microbiota and autism: Key concepts and findings. J. Autism Dev. Disord..

[71] Lopetuso LR, Scaldaferri F, Petito V, Gasbarrini A (2013). Commensal clostridia: Leading players in the maintenance of gut homeostasis. Gut Pathog..

[72] Bezawada N, Phang TH, Hold GL, Hansen R (2020). Autism spectrum disorder and the gut microbiota in children: A systematic review. Ann. Nutr. Metab..

[73] Frye RE (2015). Approaches to studying and manipulating the enteric microbiome to improve autism symptoms. Microb. Ecol. Health Dis..

[74] Lukasik J, Patro-Golab B, Horvath A, Baron R, Szajewska H (2019). Early life exposure to antibiotics and autism spectrum disorders: A systematic review. J. Autism Dev. Disord..

[75] Mirzaei R (2021). Role of microbiota-derived short-chain fatty acids in nervous system disorders. Biomed. Pharmacother..

[76] Chung WSF (2017). Prebiotic potential of pectin and pectic oligosaccharides to promote anti-inflammatory commensal bacteria in the human colon. FEMS Microbiol. Ecol..

[77] Mukherjee A, Lordan C, Ross RP, Cotter PD (2020). Gut microbes from the phylogenetically diverse genus Eubacterium and their various contributions to gut health. Gut Microbes.

[78] Abujamel TS (2022). Different alterations in gut microbiota between *Bifidobacterium longum* and fecal microbiota transplantation treatments in propionic acid rat model of autism. Nutrients.

[79] Lozupone CA, Stombaugh JI, Gordon JI, Jansson JK, Knight R (2012). Diversity, stability and resilience of the human gut microbiota. Nature.

[80] Kim, N. Sex difference of gut microbiota. *Sex/Gender-Specific Medicine in the Gastrointestinal Diseases* 363–377 (2022).

[81] Willsey HR, Willsey AJ, Wang B, State MW (2022). Genomics, convergent neuroscience and progress in understanding autism spectrum disorder. Nat. Rev. Neurosci..

[82] West KA (2022). Multi-angle meta-analysis of the gut microbiome in autism spectrum disorder: A step toward understanding patient subgroups. Sci. Rep..

