# Supplementary material for: A robust microbiome signature for autism spectrum disorder across different studies using machine learning
Source: Sci Rep. 2024 Jan 8;14:814. doi: 10.1038/s41598-023-50601-7 (PMC10774349; doi:10.1038/s41598-023-50601-7)
Supplement: Supplementary file 1 — Supplementary Figures. [file 41598_2023_50601_MOESM1_ESM.docx]

**Supplementary Figure 1.** Heat-map plotting the normalized relative abundance of each ASV per individual in the discovery dataset. Heat-map generated using heatmap.py.


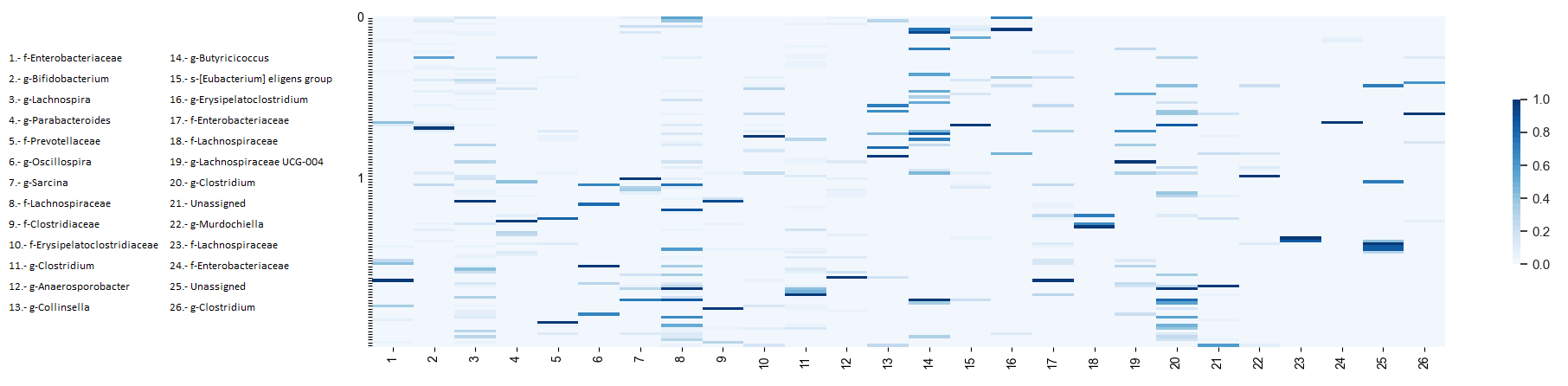


Control

ASD

**Supplementary Figure 2.** Heat-map plotting the normalized relative abundance of the identified ASVs found in PRJNA578223. Heat-map generated using heatmap.py.

Control

ASD


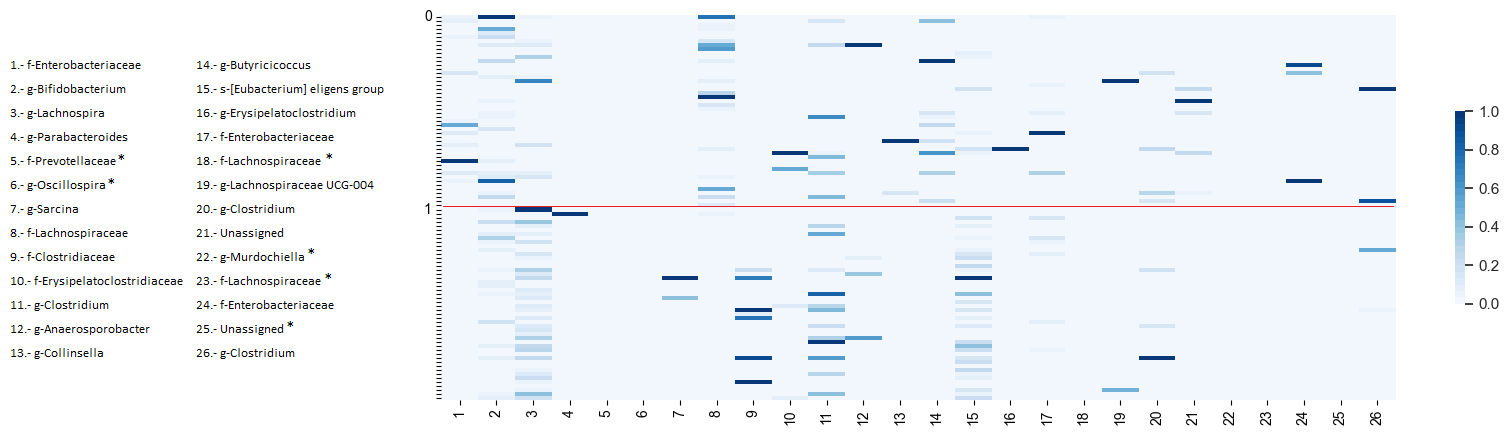


* indicates the ASVs, out of the 26 selected using REFS, that were not found in this validation dataset.

**Supplementary Figure 3.** Heat-map plotting the normalized relative abundance of the identified ASVs found in PRJNA589343. Heat-map generated using heatmap.py.


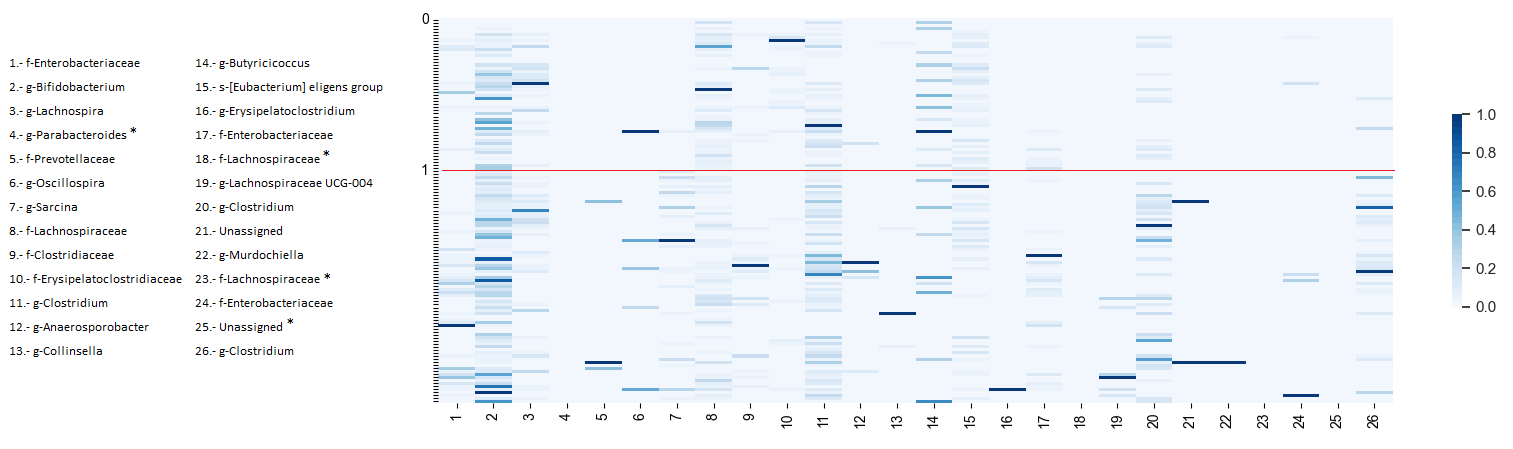


Control

ASD

* indicates the ASVs, out of the 26 selected using REFS, that were not found in this validation dataset.
